# The impact of the RTS,S malaria vaccine on uncomplicated malaria: evidence from the phase IV study districts, Upper East Region, Ghana, 2020–2022

**DOI:** 10.1186/s12936-024-05123-6

**Published:** 2024-10-10

**Authors:** Michael Rockson Adjei, Rafiq Okine, Peter Ofori Tweneboah, Janet Vanessa Baafi, Nana Akua Afriyie, Emmanuel Akwoulo Agyigewe Teviu, Josephat Ana-Imwine Nyuzaghl, Emmanuel Kofi Dzotsi, Sally-Ann Ohene, Martin Peter Grobusch

**Affiliations:** 1 Center of Tropical Medicine and Travel Medicine, Department of Infectious Diseases, Amsterdam University Medical Centers, Location AMC, University of Amsterdam, Amsterdam, The Netherlands; 2 World Health Organization, Country Office, Accra, Ghana; 3 World Health Organization, Headquarters, Geneva, Switzerland; 4 Ghana Health Service, District Health Directorate, Odumase, Sunyani West Ghana; 5 Ghana Health Service, District Health Directorate, Oforikrom Municipality, Kumasi-Oforikrom, Ghana; 6 Ghana Health Service, Regional Health Directorate, Kumasi, Ashanti Region Ghana; 7 Ghana Health Service, Regional Health Directorate, Bolgatanga, Upper East Region Ghana; 8 Institute of Tropical Medicine, and German Center of Infectious Diseases (DZIF), University of Tuebingen, Tuebingen, Germany; 9 Institute of Infectious Diseases and Molecular Medicine, University of Cape Town, Cape Town, South Africa; 10 Centre de Recherches Médicales en Lambaréné (CERMEL), Lambaréné, Gabon; 11 Masanga Medical Research Unit, Masanga, Sierra Leone

**Keywords:** Uncomplicated  malaria, Ghana, Kasena Nankana disticts, Routine surveillance data, RTS,S, Malaria vaccine

## Abstract

**Background:**

The RTS,S malaria vaccine has been prequalified for use in endemic settings prioritizing areas with moderate to high disease transmission. The impact of a vaccine at the population level may differ from observations during clinical trial due to programmatic, and individual-related factors, among others. The objective of this study was to assess the impact of the RTS,S malaria vaccine on uncomplicated malaria among children aged 12–59 months in the Phase IV study districts, Upper East Region, Ghana.

**Methods:**

A retrospective study was conducted using routine malaria surveillance data for the period 2020–2022. The burden of uncomplicated malaria was compared between the implementing (Kasena Nankana East and West districts) and comparator areas (Builsa North and South districts). The impact of RTS,S malaria vaccine was assessed by estimating the percentage reduction in uncomplicated malaria and incidence averted in the implementing area, accounting for the effect of confounders.

**Results:**

Over 50,000 episodes of uncomplicated malaria among children aged 12–59 months were included in the study. Uncomplicated malaria was reduced by 33% (95%CI 29–36) over the entire study period, but the malaria incidence averted declined from 324/1,000 (95% CI 298–339; p < 0.0001) in 2020 to 287/1000 (95% CI 274–299; p < 0.0001) in 2022.

**Conclusion:**

The RTS,S malaria vaccine significantly reduced the burden of uncomplicated malaria among children aged 12–59 months in the implementing area. The sequential marginal declines in malaria incidence averted over the study period might be due to waning of protective immunity and acquisition of natural immunity as children age. Strengthening uptake of the currently recommended vaccines and other malaria control interventions is required to improve public health impact.

## Background

Globally, there were estimated 249 million malaria cases in 2022 compared with 244 million in 2021. Majority (95%) of the cases were recorded in the World Health Organization (WHO) African Region with four countries (Nigeria, Democratic Republic of Congo, Uganda, and Mozambique) accounting for almost half of the global cases [1]. Despite the increase in cases, global malaria deaths have declined steadily from 29 per 100,000 in 2000 to 14.3 per 100,000 in 2022. Although malaria-related mortality decreased by about 61% within the same period in the African Region, children under five years were among the most vulnerable and accounted for about 80% of malaria-related mortalities globally [1].

In Ghana, malaria occurs throughout the year, and about 30% and 23% of outpatient and inpatient hospital attendances, respectively, are due to malaria [2]. In 2022, the country recorded an estimated 5.3 million malaria cases and 11,557 deaths [3]. In the Upper East Region, 452,441 malaria cases were recorded in 2022 (Ghana Health Service, District Health Information Management System, 2022; unpublished).

In May 2015, the Global Technical Strategy (GTS) was adopted by the World Health Assembly as a comprehensive framework to guide countries in their efforts to accelerate malaria elimination. The strategy sets the target of reducing global malaria incidence and mortality by at least 90%, respectively, by 2030 [4]. The GTS is threatened by changes in disease epidemiology including emergence of insecticide-resistant mosquito species and drug-resistant *Plasmodium* strains [1]. Again, preferences and equity issues limit availability and use of the existing control interventions, resulting in disparities in malaria burden globally [5]. Malaria vaccine has the potential of mitigating the emergence of drug-resistant *Plasmodium* species by limiting the use of anti-malarial drugs [6].

Given that children are the most vulnerable but reliant on adults for day-to-day implementation of malaria control interventions (for example the mounting of ITNs every night), expanding access to self-enabling interventions could complement efforts in reducing the disease burden among this group. The malaria vaccine is one such intervention and wields prospect of further reducing the malaria-related mortality among children [4]. During Phase-III clinical trial, the RTS,S malaria vaccine was found to reduce the burden of clinical malaria by 28.3% (95% CI 23.3–32.9), and 36.3% (95% CI 31.8–40.5) in the 3-dose and dose 4 groups, respectively, during a median follow-up period of 46 months [7].

The RTS,S malaria vaccine was introduced into routine immunization in 42 districts in seven high-burden regions of Ghana in May 2019 as part of the pilot, to assess its feasibility, safety, and impact before recommendation for wider use [8]. Following the results from the pilot (in Ghana, Kenya, and Malawi), the WHO recommended the broader use of the vaccine for the prevention of *P. falciparum* malaria in children living in endemic areas, in October 2021 [9].

Vaccine efficacy studies are carried out under stringent conditions and the estimates represent vaccine performance under ideal conditions [10]. However, real-life vaccine performance may differ from observations during clinical trials due to programmatic, and individual-related factors, among others. The objective of this study was to assess the impact of the RTS,S malaria vaccine on uncomplicated malaria episodes among children 12–59 months in the Upper East Region of Ghana, using routine malaria surveillance data.

## Methods

### Study design

A retrospective study was conducted in the Kasena Nankana and Builsa districts in the Upper East Region using routine malaria surveillance data from the District Health Information Management System (DHIMS-2) for the period 2020–2022. The implementation and comparator areas were Kasena Nankana and Builsa districts, respectively.

### Study setting

**Fig. 1:**
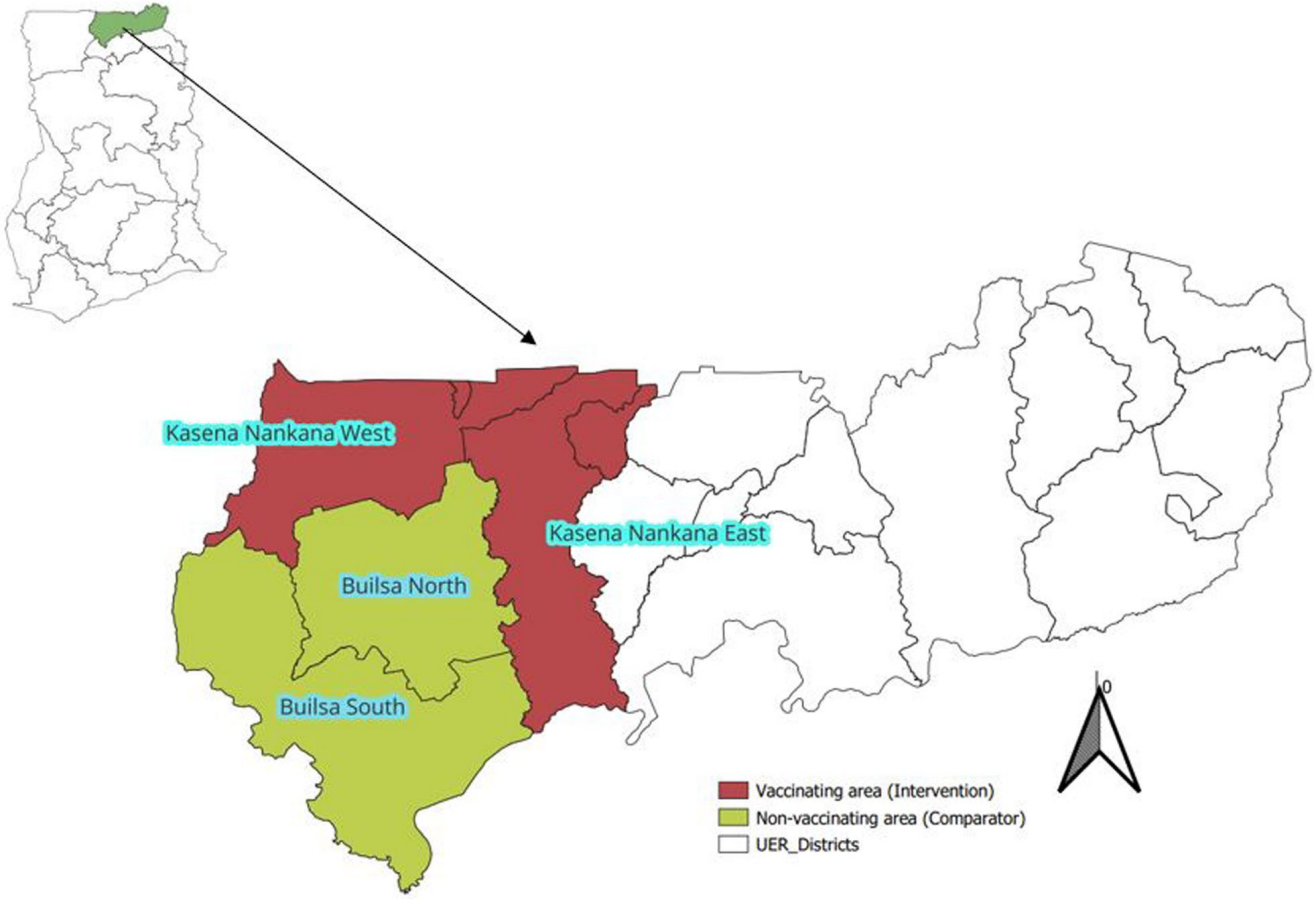
Map of study area and distribution of key malaria control interventions in the study area, Upper East Region, Ghana; 2020–2022

The Upper East Region is situated in the north-eastern corner of Ghana. It shares borders with Burkina Faso to the north, Togo to the east, the North East Region to the south, and the Upper West Region to the west (Fig. 1). The region occupies a land mass of 8842 square kilometres and constitute 2.7% of the total landmass of Ghana [11]. It has 15 administrative districts and 101 sub-districts. The projected population from the 2021 census was 1,318,523, with children 12–59 months constituting 16% [11]. There are 352 health facilities comprising 20 hospitals (7 public, 3 faith-based, and 10 private), 67 health centres, 41 clinics and maternity homes, and 224 community-based health planning and services (CHPS) compounds [12].

The land is relatively flat, with a few hills to the East and Southeast. Most of the eastern and western parts of the region lie within the basins of the Volta River and its tributaries. Upper East has a tropical wet and dry or savanna climate with yearly temperature of 31.47 ºC (88.65 ºF) which is 2.6% higher than Ghana’s averages [11]. It has one rainfall season that begins in May, peaks in August, and lasts until September and typically receives about 44.5 mm (1.75 in.) of precipitation annually [13, 14].

The study areas were purposively selected because they participated in the GSK-led Phase IV studies and were, therefore, likely to have high quality routine malaria surveillance data to facilitate the study. The Phase-IV studies were instituted to monitor adverse events reported in the paediatric population following introduction of the malaria vaccine, by comparing occurrence in the implementing area (Kasena Nankana districts) and the comparator (Builsa districts) [15]. Because the districts were excluded from the evaluation studies that accompanied the pilot programme and the post-introduction evaluation (PIE) [8], this study which is parallel to the Phase-IV studies presented the opportunity to assess the impact of the pilot programme, specifically impact on uncomplicated malaria, since this was not evaluated in the malaria vaccine implementation programme (MVIP) or the Phase-IV studies in the region.

The Kasena Nankana West and East districts (the intervention area) are located in the north-western part of the Upper East Region with the east district stretching southwards and sharing border with North East Region (Fig. 1). Both districts share boundaries with the Builsa North and South districts, the comparator area. The total population from the 2021 population and housing census was 190,630 (Kasena Nankana West—90,735; Kasena Nankana East—99,895) with growth rate of 2% [16]. There are 30 health facilities in Kasena Nankana East comprising one hospital, two health centres, 24 community-based health planning and services (CHPS) compounds, two private clinics, and one faith-based clinic. Kasena Nankana West has 63 health facilities comprising one hospital, seven health centres, 52 CHPS compounds, and three private clinics. The districts were the only vaccinating areas in the region during the MVIP [8, 17].

Builsa North and South districts are in the south-western part of the Upper East Region. The total population was 56,571 for Builsa North, and 36,575 for Builsa South [16]. Builsa North District has 15 health facilities comprising one hospital, three health centres, nine CHPS compound, one private clinic, and one faith-based clinics. Builsa South has 16 health facilities comprising two health centres, and 14 CHPS compounds. Apart from sharing similar health-seeking behaviour and socio-cultural practices (Ghana Health Service, Upper East Regional Health Directorate, Annual Report, 2020, unpublished), the Builsa districts (as it was with the Kasena Nankana districts) benefitted from capacity building and logistics support during the Phase-IV studies to harmonize healthcare practices and data management [15], making them comparable with the intervention districts (except for implementation of malaria control measures).

### Implementation of malaria prevention interventions

There is intense seasonal transmission of malaria in the entire region with peak period coinciding with the rainy season due to conducive environmental conditions for proliferation of the vectors, *Anopheles* mosquito species [14]. Implementation of malaria prevention interventions (with impact on health of children aged 12–59 months) varies across the region. While continuous distribution of ITN and seasonal malaria chemoprevention (SMC) were implemented in all districts of the region, indoor residual spraying (IRS) and larval source management (LSM) were limited to specific areas (Table 1) in accordance with the selection criteria of the National Malaria Elimination Programme (NMEP) [17].

**Table 1:** Uptake of other malaria control interventions in the study areas, Upper East Region, Ghana; 2020–2022

District	Malaria intervention											
	Coverage (%) of indoor residual spraying			Coverage (%) of larval source management			Coverage (%) of ITN distribution			Mean number of antimalaria drug doses/child for seasonal malaria chemoprevention		
	2020	2021	2022	2020	2021	2022	2020	2021	2022	2020	2021	2022
Kasena Nankana East	0^a^	0^a^	0^a^	100	100	100	93.1	92.8	97.7	3.0	3.7	3.9
Kasena Nankana West	0^a^	0^a^	100	0^a^	0^a^	0^a^	92.8	90.3	95	3.3	3.6	3.9
Builsa North	0^a^	100	100	0^a^	0^a^	0^a^	94.2	98.6	96.4	3.6	4.0	4.0
Builsa South	0^a^	0^a^	100	0^a^	0^a^	0^a^	94.5	97.1	95.4	4.0	4.0	4.0

^a^O = No implementation of intervention

The average coverage of continuous ITN distribution (2020–2022) was 93.5% for the Kasena Nankana districts (Upper East Region, DHIMS-2, unpublished). For SMC, the average number of doses administered to children aged 12–59 months per season in the districts was 3.6 out of four (Upper East Region, DHIMS-2, unpublished). IRS was implemented in Kasena Nankana West in 2022 and 100% of the targeted population were protected (Upper East Region, DHIMS-2, unpublished). LSM was deployed in Kasena Nankana East for the entire study period, and all 325 targeted potential mosquito breeding sites were covered (Upper East Region, activity reports, 2020–2022 unpublished).

An average of 96% of the target population (children receiving second dose of measles-rubella vaccine, and antenatal registrants) in the Builsa districts received ITN through continuous distribution over the study period (Upper East Region, DHIMS-2, unpublished). Children aged 12–59 months received an average of four doses of anti-malarial drugs for the SMC campaigns conducted in the districts (Upper East Region, DHIMS-2, unpublished). Builsa North conducted IRS in 2021 and 100% of the targeted population were covered. In 2022, both districts implemented IRS and all targeted persons were protected (Table 1).

### Data collection

Study data including population of children aged 12–59 months, RTS,S malaria vaccine doses administered, malaria episodes (suspected, tested, and confirmed), report completeness rates, and ITN and SMC coverages were extracted from the DHIMS-2 platform; while the coverages for IRS and LSM were retrieved from activity reports.

DHIMS-2 is free open-source software (https://www.dhis2.org) developed from the district health information system (DHIS). It was first introduced in Ghana in the year 2007 and was primarily used for collecting and reporting health data. Data is collected at the health facility level using standard registers, collated onto summary forms, and entered into the DHIMS-2 platform weekly or monthly. The interface is password protected and accessible only by authorized healthcare managers.

The extracted data was verified from the sources (paper-based reports and registers) and validated for completeness and consistency before exporting for analysis.

### Data analysis

**Fig. 2:**
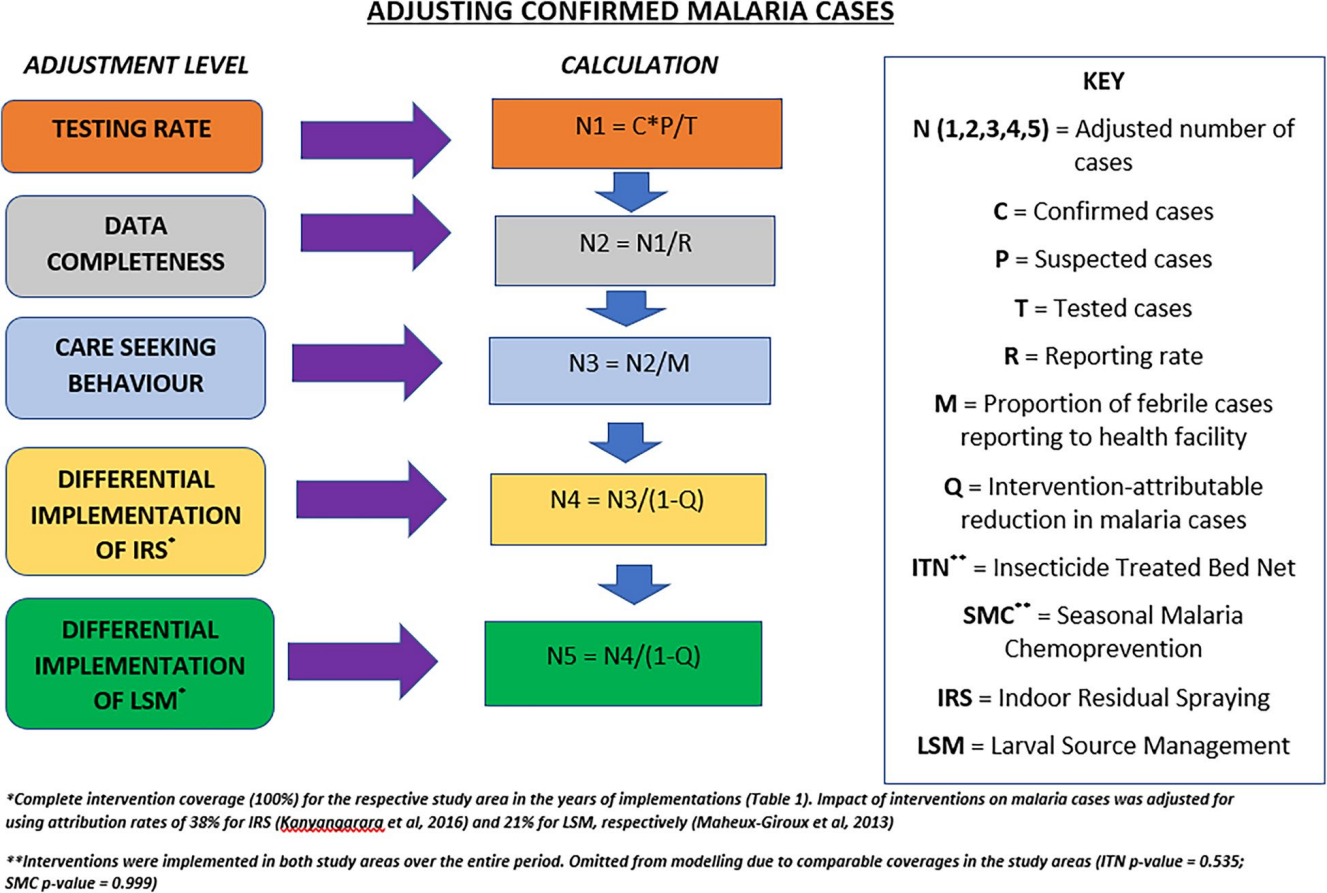
Schema for adjusting laboratory confirmed malaria cases, Upper East Region, Ghana; 2020–2022

Data was exported into Epi Info statistical software (Epi Info version 7.2.2.16, www.cdc.gov.epiinfo) for analysis. Adjustment was made for the effect of confounders including health seeking behaviour (proportion of febrile cases reporting to health facilities), health care practice (testing and reporting rates), and implementation of district-specific malaria control interventions on the episodes of malaria recorded by the study sites (Tables 1 and 2 and Fig. 2). Only laboratory confirmed uncomplicated malaria episodes were entered into the final analysis, and data from all public health facilities (115/124; 92.7%) in the study areas were included. Attributable rates for IRS (38%) and LSM (21%) were adopted from studies conducted elsewhere in the African Region [18, 19]. However, adjustments were not made for ITN and SMC implementation due to comparable coverages in the study sites (ITN p-value = 0.535; SMC p-value = 0.999).

**Table 2:** Adjustment parameters by study districts, Upper East Region, Ghana; 2020–2022

District	HSB (%)			MTR (%)			RCR (%)		
	2020	2021	2022	2020	2021	2022	2020	2021	2022
Kasena Nankana East	78	75	75	100	99.8	100	100	91	95
Kasena Nankana West	76	77	75	100	100	100	96	84	92
Builsa North	75	78	74	98.7	99.9	100	96	94	77
Builsa South	79	74	76	100	100	100	99	99	99

*HSB* health seeking behaviour, *MTR* Malaria testing rate, *RCR* report completeness rate

The RTS,S vaccination coverages for the respective doses and study periods were calculated by dividing the number of children receiving a particular dose in a specific year with the population of surviving infants, multiplied by 100. The dropout rates were estimated by dividing the difference between doses administered for the respective vaccine schedules with doses administered for preceding schedules, multiplied by 100 [20].

The malaria incidence rate ratio, IRR (comparing incidence rate in the implementing area with the comparator) was computed. The impact of the RTS,S malaria vaccine at the population level was determined by estimating the percentage reduction in uncomplicated malaria episodes, calculated by subtracting the IRR from 1 and multiplied by 100 [21]. Results were computed at 95% confidence level for unadjusted and adjusted estimates and presented as text, tables, and graphs.

## Results

**Fig. 3:**
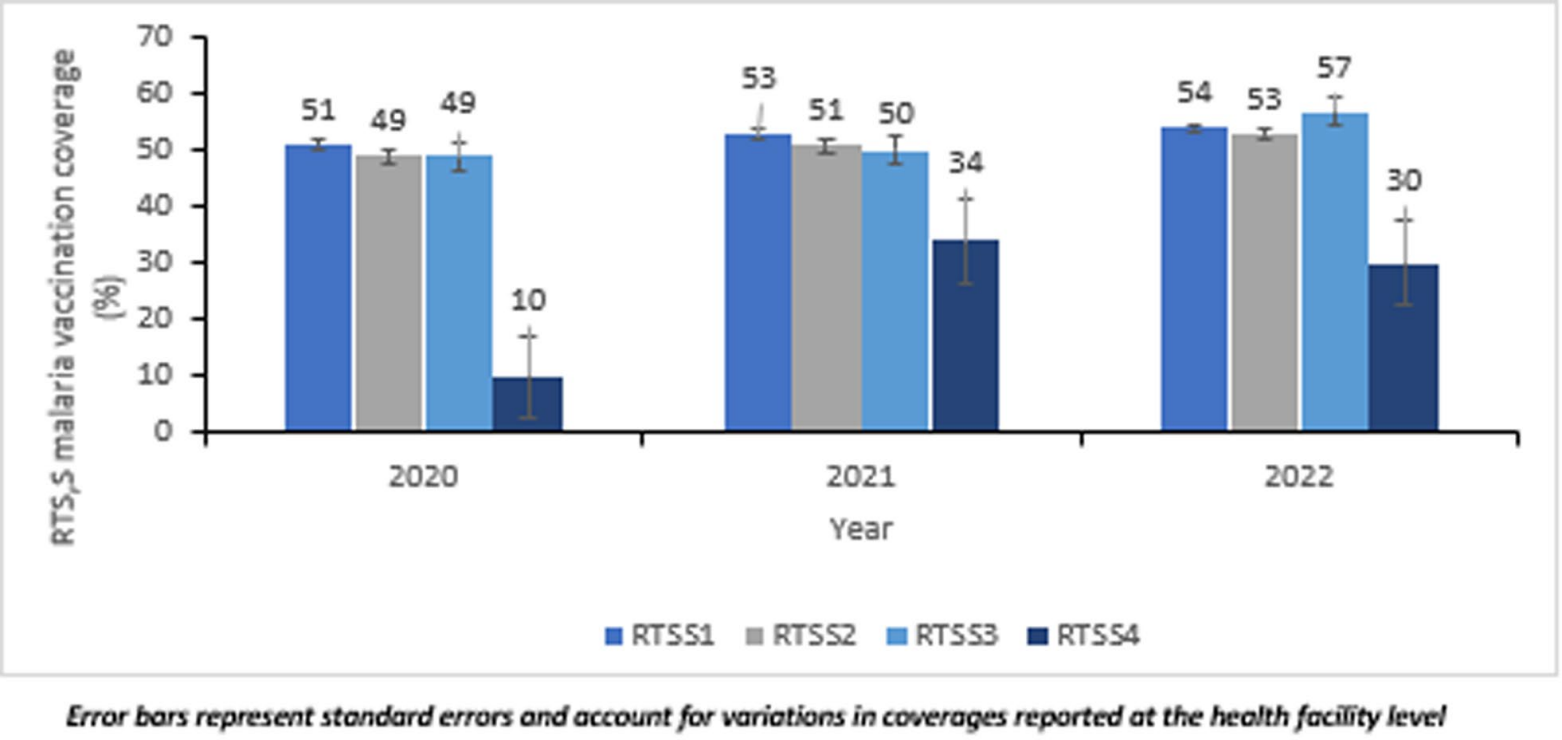
Trend of RTS,S malaria vaccination coverage, Upper East Region, Ghana; 2020–2022

A total number of 48,958 doses of the RTS,S malaria vaccine was administered in the vaccinating area—Kasena Nankana East and West districts—over the study period. Approximately 51% of the doses was administered in Kasena Nankana East district. Nearly 29% of the total doses was administered in 2020, 35% in 2021, and 36% in 2022. Coverages of the respective schedules increased marginally over the study period, except for the fourth dose, which increased more than three-folds from 10% in 2020 to 34% 2021 before declining to 30% in 2022 (Fig. 3). The average  dropout rate over the study period was 1.6% for RTS,S1/3, 52.8% for RTS,S1/4, and 52% for RTS,S3/4, respectively.

Fifty-four thousand, five hundred and seventy-two (54,572) uncomplicated malaria episodes (observed) were recorded during the study period. Approximately 59% (32,274) were recorded in the implementing area (Kasena Nankana East and West), and out of which 59.6% (19,296) were from Kasena Nankana West District alone. About 51.2% (11,437) of the episodes observed in the comparator area were recorded in Builsa South. Following the adjustment, a total of 96,793 expected episodes of uncomplicated malaria (57.1% from the implementing area) were estimated.

**Fig. 4:**
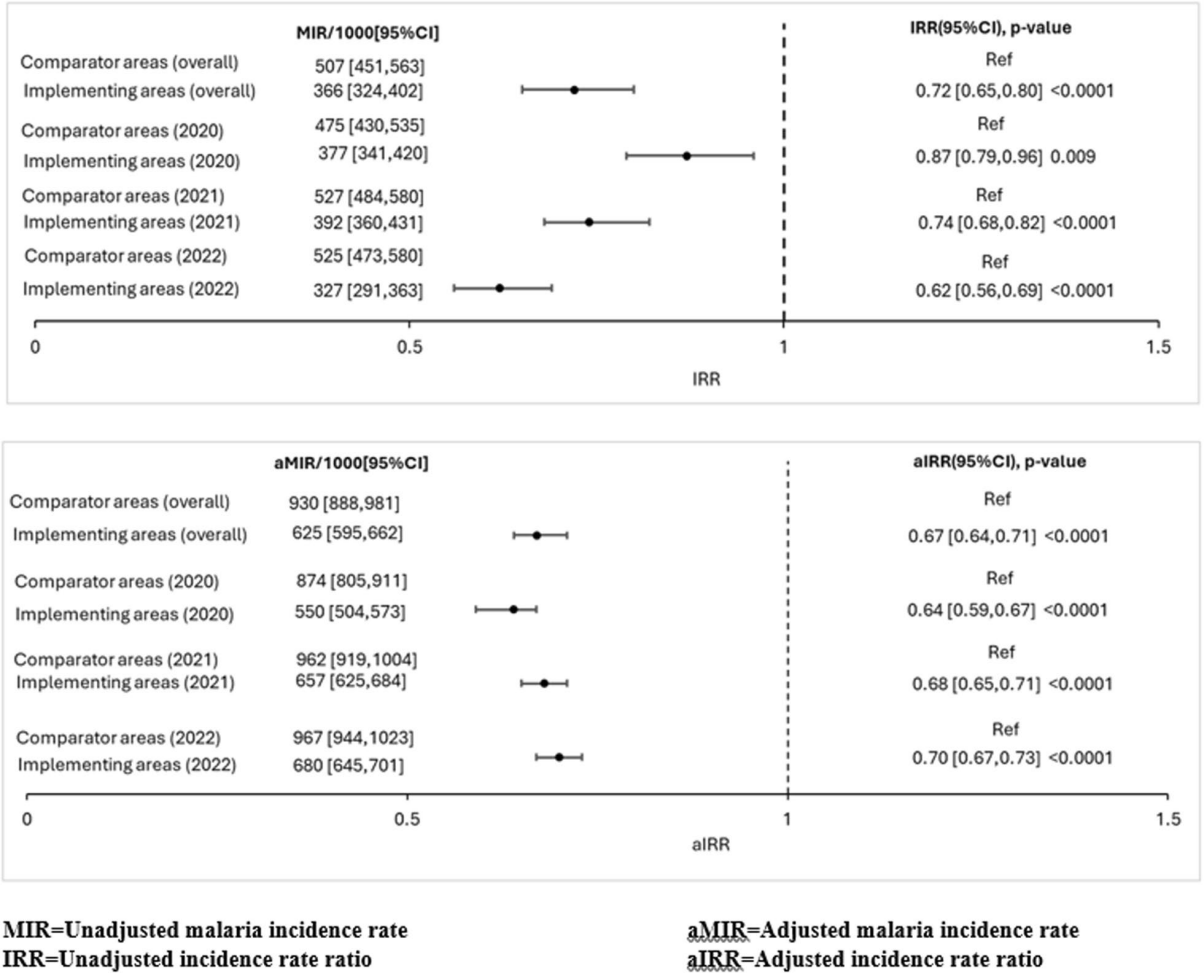
Malaria incidence and incidence ratios, MVIP impact study, Upper East Region, Ghana; 2020–2022

Uncomplicated malaria episodes reduced by 28% per year in the implementing area compared with the comparator area and the magnitude of reduction increased from 13% in 2020 to 38% in 2022. Adjusting for effect of confounders, uncomplicated malaria episodes reduced by 33% (95% CI 29–36) in the implementing area, but malaria incidence averted declined over the study period from 324/1000 (95% CI 298–339; p < 0.0001) in 2020 to 305/1000 (95% CI 291–318; p < 0.0001) in 2021, and 287/1000 (95% CI 274–299; p < 0.0001) in 2022 (Fig. 4).

## Discussion

Ghana adopted a phased approach in the malaria vaccine introduction with an initial schedule of 6, 7, 9, and 24 months [22, 23]. The study assessed the impact of the RTS,S malaria vaccine on uncomplicated malaria episodes among children aged 12–59 months. The rationale for the selection of the study population was that the protective efficacy of RTS,S vaccine is significant after administration of the third dose [7], and most eligible children would have taken the first three doses by age 12 months.

The trend of vaccine uptake reflects the pathway of the introduction process in the region and the country at large. Profound vaccine hesitancy was encountered in the early phase, and this resulted in low demand which improved over time with strengthening of stakeholder engagement and community education [24]. The high fourth-dose dropout rate (RTS,S1/4 and RTS,S3/4) might have occurred because the schedule fell outside the regular childhood immunization platform, and caregivers needed to make further visits to complete the vaccination.

Additionally, while some caregivers might have relocated to non-vaccinating areas before the due date of the subsequent doses, the waiting interval, particularly between the third and fourth doses might have contributed to appointment failures due to forgetfulness [8]. To address the challenges, health workers sent reminders to caregivers and embarked on defaulter tracing to catch-up on missed children [24], but implementation of these strategies were impacted by competing activities, especially the conduct of multiple COVID-19 vaccination campaigns.

From the study, there was 33% (95% CI 29–36) reduction in uncomplicated malaria episodes in the implementing area. Although the malaria incidence averted declined over time, this was not significant, given the overlapping confidence intervals of the estimates for the respective years. Nonetheless, this finding is consistent with the observation that RTS,S malaria vaccine efficacy is reasonably high over six months following completion of the first three doses, and wanes thereafter [25]. Protective efficacy is restored after the fourth dose, but not to the same level as seen after the initial three doses, possibly due to acquisition of natural infection [25]. Additionally, the acquisition of natural immunity as children age results in relatively low incidence of clinical malaria among older children, contributing to the decline in the malaria incidence averted over time, as observed from the study [26].

In the unadjusted estimates, the malaria incidence averted increased progressively over the study period, possibly due to the complementary effect of the other malaria control interventions [27]. The observation highlights the need to strengthen the use of other malaria control measures in malaria vaccine implementing areas to sustain the overall impact on disease burden.

The study is not without limitations. The applicability of the population level estimates at individual level could not be ascertained due to the nature of the data used in the study—aggregate data. Further, only data from facilities reporting in DHIMS-2 were used for the study. Data from private health facilities, community pharmacies and alternative practitioners were unavailable. To mitigate this, the number of confirmed malaria episodes were adjusted for health-seeking behaviour, assuming all patients accessed care from public health facilities. Again, the possibility of inherent data quality issues impacting reliability and validity of the findings could not be ruled out completely, although data verification, validation, and adjustment were conducted. Additionally, the likelihood of contamination (due to malaria vaccine being received by children in the comparator area or non-eligible age group in the intervention area [8]) diluting estimate of impact cannot be underestimated. Furthermore, the impact of the vaccine on severe malaria was not assessed due to inadequate data. Lastly, attribution rates for IRS, and LSM for the study areas, respectively, could not be estimated due to incomplete data. The application of attribution rates from other studies could impact reliability of the results. 

## Conclusion

The study assessed the impact of routine use of RTS,S malaria vaccine on uncomplicated malaria among children aged 12–59 months. The vaccine reduced episodes of uncomplicated malaria by 33%. Although the malaria incidence averted decreased marginally over time, the impact on uncomplicated malaria remained significant. Given that, this substantial impact was achieved in the context of moderate coverage of the primary vaccine series and low fourth dose coverage, strengthening uptake of the currently recommended vaccines and other malaria control interventions could improve overall public health impact.

Caregiver education and institution of robust defaulter tracing mechanism would improve continuity of uptake, strengthen consistent use of other malaria control measures, and reduce drop-out rates especially for the fourth dose. Additionally, countries planning introduction should consider aligning the fourth vaccine dose with other second year of life (2YL) vaccines or health interventions, as has been done by Ghana since 2023, to improve uptake and reduce dropout rate. Lastly, although the fourth dose restores protective efficacy and may last longer, administration of a fifth dose should be considered one year after the fourth in areas where malaria risk remains significant to improve protective effectiveness [25].

## Data Availability

Data will be made available upon reasonable request.

## Abbreviations

CHPS: Community-based Health Planning and Service
DHIMS: District Health Information Management System
DHIS: District Health Information Management System
GSK: GlaxoSmithKline
IRS: Indoor residual spraying
ITN: Insecticide-treated bed net
LSM: Larval source management
MVIP: Malaria Vaccine Implementation Programme
NMEP: National Malaria Elimination Programme
PIE: Post-introduction evaluation
SMC: Seasonal malaria chemoprevention
2YL: Second year of life
